# Population genetic differentiation of the hydrothermal vent crab *Austinograea alayseae* (Crustacea: Bythograeidae) in the Southwest Pacific Ocean

**DOI:** 10.1371/journal.pone.0215829

**Published:** 2019-04-24

**Authors:** Won-Kyung Lee, Se-Joo Kim, Bo Kyeng Hou, Cindy Lee Van Dover, Se-Jong Ju

**Affiliations:** 1 Global Ocean Resources Research Center, Korea Institute of Ocean Science & Technology, Busan Metropolitan City, Republic of Korea; 2 Genome Editing Research Center, Korea Research Institute of Bioscience and Biotechnology, Daejeon, Republic of Korea; 3 Division of Marine Science and Conservation, Nicholas School of the Environment, Duke University, Beaufort, NC, United States of America; 4 Marine Biology Major, University of Science & Technology, Daejeon, Republic of Korea; Florida Institute of Technology, UNITED STATES

## Abstract

To understand the origin, migration, and distribution of organisms across disjunct deep-sea vent habitats, previous studies have documented the population genetic structures of widely distributed fauna, such as gastropods, bivalves, barnacles, and squat lobsters. However, a limited number of investigations has been conducted in the Southwest Pacific Ocean, and many questions remain. In this study, we determined the population structure of the bythograeid crab *Austinograea alayseae* from three adjacent vent systems (Manus Basin, North Fiji Basin, and Tonga Arc) in the Southwest Pacific Ocean using the sequences of two mitochondrial genes (*COI* and *16S rDNA*) and one nuclear gene (*28S rDNA*). Populations were divided into a Manus clade and a North Fiji–Tonga clade, with sequence divergence values in the middle of the barcoding gap for bythograeids. We inferred that hydrographic and/or physical barriers act on the gene flow of *A*. *alayseae* between the Manus and North Fiji basins. *Austinograea alayseae* individuals interact freely between the North Fiji Basin and the Lau Basin (Tonga Arc). Although further studies of genetic differentiation over a geological time scale, life-history attributes, and genome-based population genetics are needed to improve our understanding of the evolutionary history of *A*. *alayseae*, our results contribute to elucidating the phylogeny, evolution, and biogeography of bythograeids.

## Introduction

Hydrothermal vent environments are characterized by a lack of light (aside from that generated by high-temperature fluids [1]), lack of photosynthesis, high pressure, steep temperature gradients, and high levels of metals and dissolved gases [2–4]. Since the discovery of hydrothermal vents along the Galapagos Rift in 1977, the description of new species has progressed steadily, and new hydrothermal vent sites have been discovered in deep areas of the seafloor. Seven hundred valid species had been described by 2011, and description of additional species continues [5]. Phylogenetic evidence based on molecular markers generally supports traditional taxonomy, and sometimes reveals the existence of sibling (or cryptic) species [6–8]. However, the origin, migration, and distribution of vent fauna across disjunct vent habitats that may undergo repetitive creation and extinction cycles at a time scale similar to the generation times of vent species are not yet fully understood.

Hydrological and physical processes are the most important factors leading to allopatric speciation in the deep-sea [9–11]. Additionally, in hydrothermal vent ecosystems, the continuity or discontinuity of ocean circulation, tectonic history, seawater temperature, and hydrothermal fluid components strongly influence the biogeographic distribution of vent fauna [12]. Based on biogeographic relationships, genetic pools, phylogenetic analyses of vent species, and larval dispersal modeling, approximately 700 known volcanic areas on the deep-sea floor can be clustered into 11 biogeographic provinces [13–15]. Previous genetic studies have noted that the populations of species in vent fields within back-arc basins are well connected, although basin-to-basin transport is impeded by dispersal barriers and directionality [7, 15, 16].

Large hydrothermal vent areas in the Southwest Pacific Ocean are defined as belonging to the single “South-West Pacific Area” biogeographic province, which consists primarily of the Manus Basin, the North Fiji Basin, and the Lau/Tonga region [13, 14]. Although the vent communities in the three regions are visually similar, biogeographic relations indicate that the North Fiji Basin and Lau/Tonga regions are most closely related [17]. Additionally, based on previous geological studies, volcanic areas spanning from North Fiji to Lau/Tonga have been proposed to originate from the disruption of a single arc [11, 18, 19]. To elucidate the genetic flows and distribution patterns of hydrothermal vent fauna among basins, the biodiversity and population genetic structures must be understood at a large spatial scale. To date, investigations in these areas are less well documented and many questions remain [15, 20, 21] (Table 1).

**Table 1:** Summary of the population structure of hydrothermal vent organisms living in the Southwest Pacific Ocean. Different shade indicates population differentiation based on *cytochrome oxidase subunit I* (*COI*) sequences of individuals from distinct geographical regions.

Phylum	Species	Region			References
		Manus	N. Fiji	Tonga/Lau	
Arthropoda	*Munidopsis lauensis*		-		[7]
	*Chorocaris* sp. 2			-	[7]
	*Eochionelasmus ohtai*		-	-	[22]
	*Vulcanolepas* cf. *parensis*		-	-	[22]
Mollusca	*Olgasolaris tollmanni*		-		[23]
	*Bathymodiolus manuensis*		-	-	[24]
	*Ifremeria nautilei*				[16, 25]

-, indicates unavailable or undiscovered samples or no data.

The distribution of the decapod family Bythograeidae Williams, 1980, is restricted to hydrothermal vents (or in close proximity to vents), and these species are typically considered to be omnivorous predators in back-arc basin vent communities [17, 26, 27]. Based on morphological taxonomy and molecular phylogenetics, this family is recognized as a sister taxon of the superfamily Xanthoidea, containing 16 species in six genera, including two recently described *Austinograea* species [28, 29–33]. Most bythograeid species occur in the Pacific Ocean; only two species, *Austinograea rodriguezensis* (Indian Ocean) and *Segonzacia mesatlantica* (Atlantic Ocean), are known outside of the Pacific. In particular, all species in the genus *Austinograea* except *A*. *rodriguezensis* colonize hydrothermal vents in the western Pacific Ocean [17, 33, 34]. The factors that drove the accelerated adaptive radiation and speciation of *Austinograea* in the Pacific Ocean remain unknown. The examination of phylogenetic relationships among *Austinograea* species could help to clarify the interaction between biological evolution and geographical processes at deep-sea hydrothermal vents. Population genetic analyses could contribute to our understanding of the larval dispersal capacity [35–37], but genetic analysis of bythograeids at the population level has been performed in only one species, *A*. *rodriguezensis*.

*Austinograea alayseae* Guinot, 1990, is distributed widely in hydrothermal vent fields from Manus to Lau/Tonga in the Southwest Pacific Ocean [17, 34, 38]. The origin, migration, adaptation, and genetic structure of this species in the Southwest Pacific Ocean are unclear, despite previous studies of its phylogeny using DNA barcoding and mitochondrial genomics [17, 34, 39]. In this study, we obtained two mitochondrial sequences and one nuclear gene sequence of *A*. *alayseae* collected from hydrothermal vents in the Manus and North Fiji basins and the Tonga Arc, and confirmed the phylogenetic position of this species within the Bythograeidae. We also identified population genetic divergence and migration events of *A*. *alayseae*, and discuss the biogeographic connections among these three vent regions in the Southwest Pacific Ocean.

## Materials and methods

### Ethics statement

Permission for sampling in Fiji’s Exclusive Economic Zone (EEZ) was granted by the Ministry of Land and Natural Resources, Republic of Fiji, to KIOST Minerals Limited, Korea Institute of Ocean Science and Technology. The permit for the collection of biological samples in Tonga’s EEZ was issued by the Minister for Lands and Natural Resources, Kingdom of Tonga, to KIOST Minerals Limited, Korea Institute of Ocean Science and Technology. The license for exclusive exploration in the Manus Basin was granted by the government of Papua New Guinea to Nautilus Minerals, and biological samples were loaned to Duke University for scientific research.

### Vent crab sampling and identification

**Fig 1:**
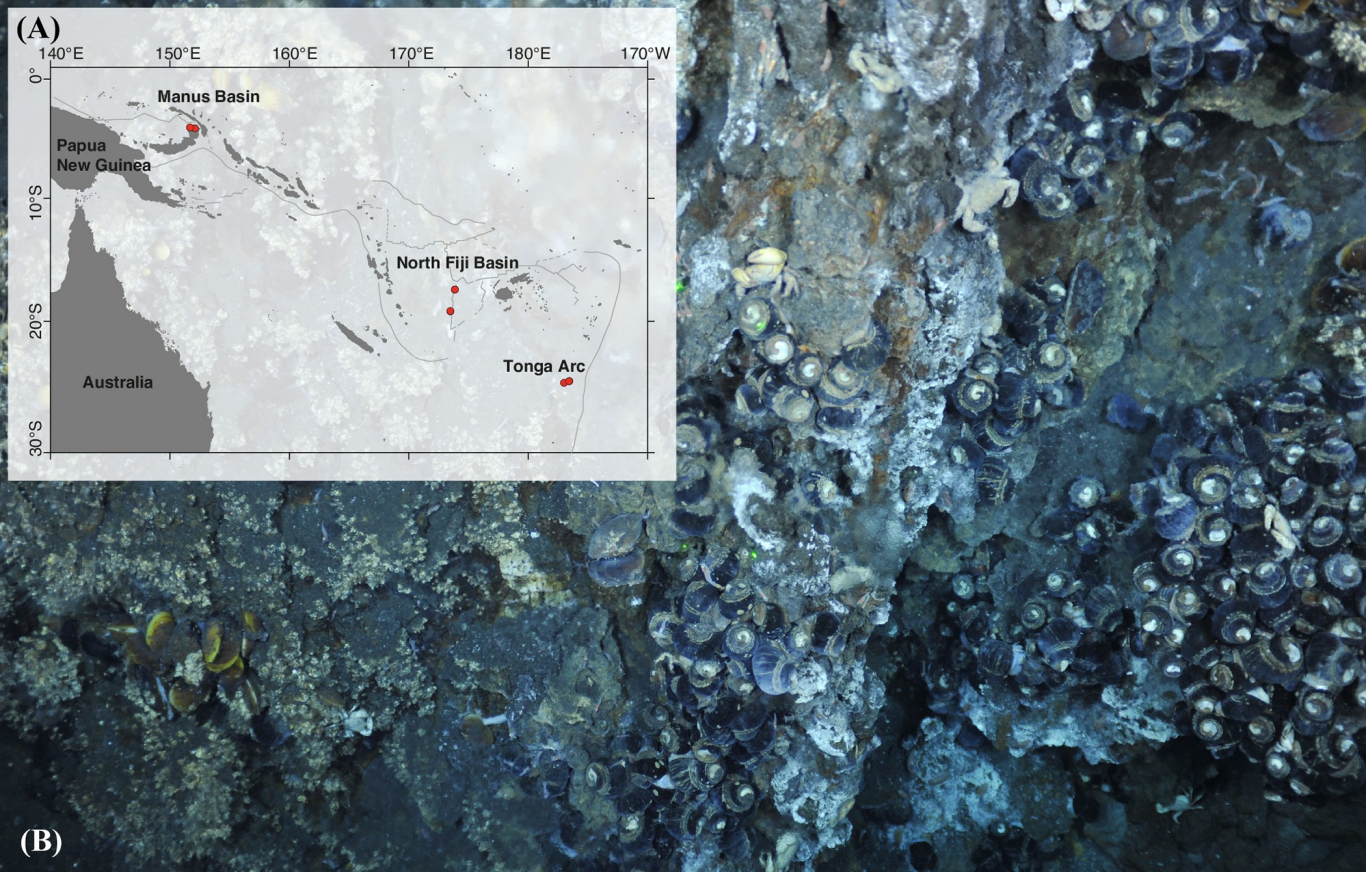
Map of *A*. *alayseae* sampling locations in the Southwest Pacific Ocean and a photograph of a hydrothermal vent in the North Fiji Basin and its associated biological communities. (A) Sampling locations of *A*. *alayseae*. Lines represent subduction zones. Red circles indicate sampling areas. Some sampling sites located short distances (<5 km) from each other (R1968-C6 and R1970-C3, R1964-C1 and 1966-C11, TA25A and TA25D) are marked with single circle. (B) An active vent at the 1970-C3 sampling site in the North Fiji Basin. The dominant visible taxa in the image are the provannid snail *Ifremeria nautilei* (center bottom and right) and the balanomorph barnacle *Eochionelasmus ohtai* (left). A small cluster of mytilid mussels, *Bathymodiolus* sp., and alvinocaridid shrimp species are visible. Scattered individuals of the bythograeid crab *A*. *alayseae* are generally seen in association with *I*. *nautilei* and *E*. *ohtai*.

Specimens of bythograeid crabs were collected using suction samplers mounted on remotely operated vehicles from twelve vent sites in the Manus Basin, the North Fiji Basin, and the Tonga Arc of the Southwest Pacific Ocean (Fig 1; Table 2). On board, all specimens were immediately preserved in 95% ethanol or stored at –80°C until genetic analysis. The specimens were identified as *A*. *alayseae*, *A*. *hourdezi*, and *Gandalfus puia* on the basis of the morphological characteristics and *cytochrome oxidase subunit I* (*COI*) DNA barcodes, following the methods used in previous studies [33, 39, 40]. Detailed information about the specimens is provided in S1 Table.

**Table 2:** Sampling information for bythograeid crabs collected from hydrothermal vent regions in the Southwest Pacific Ocean.

Region	Field ID	Number of specimens			Latitude	Longitude
		*A*. *alayseae*	*A*. *hourdezi*	*G*. *puia*		
**Manus Basin**	Solwara 1	9	-	-	3.79° S	152.09° E
	Solwara 8	1	-	-	3.73° S	151.67° E
**Fiji Basin**	R1970-C3	8	-	-	17.11° S	173.87° E
	R1959-C2	-	2	-	19.05° S	173.48° E
	R1964-C1	1	5	-	18.85° S	173.50° E
	R1966-C11	1	-	-	18.82° S	173.50° E
	R1966-C12	-	1	-	18.82° S	173.50° E
	R1968-C6	1	-	-	17.12° S	173.87° E
**Tonga Arc**	TA26A	1	-	2	24.48° S	177.00° W
	TA25A	4	-	-	24.35° S	176.57° W
	TA25C	-	-	1	24.35° S	176.54° W
	TA25D	12	-	2	24.35° S	176.57° W

### DNA extraction, PCR amplification, and sequencing

A microscopic section of muscle tissue was dissected from a pereopod of each specimen for DNA extraction. Total genomic DNA was extracted using the RED Extract-N-Amp PCR Kit (Sigma-Aldrich Co., Brooklyn, NY, USA) [41]. The partial sequences of two mitochondrial genes (*COI* and *16S rDNA*) and one nuclear gene (*28S rDNA*) were determined using previously published primers (Table 3). PCR amplification was performed in a total volume of 50 μL containing 1 μL genomic DNA, 4 μL dNTP mixture (2.5 mM each), 1 μL (10 pmol) of each primer, 5 μL 10X Ex Taq Buffer (Mg2+ plus), and 1.25 U Takara Ex Taq DNA Polymerase (Takara Biotechnology Co., Tokyo, Japan) under the following conditions: initial denaturation at 94°C for 2 min, followed by 40 cycles of denaturation (10 s at 95°C), primer annealing (30 s at 42°C in the first 5 cycles and 30 s at 48°C in the last 35 cycles for *16S rDNA*; 30 s at 53°C for *28S rDNA*), and extension (2 min at 72°C), and then a final extension (2 min at 72°C). To avoid interference from nuclear mitochondrial pseudogenes, PCR amplification for *COI* of *A*. *alayseae* was carried out according to the method of Kim et al. (2013) [34]. Finally, Sanger sequencing was conducted at Macrogen Service (Seoul, Korea) using the ABI PRISM 3730XL Analyzer (Applied Biosystems) with BigDye (R) Terminator v3.1 Cycle Sequencing kits (Applied Biosystems). Sequences obtained in this study were trimmed and annotated using Geneious Prime (Biomatters, Auckland, New Zealand), then adjusted through visual inspection. The newly obtained sequences of *A*. *alayseae*, *A*. *hourdezi*, and *G*. *puia* (S1–S3 Tables) were registered in GenBank.

**Table 3:** Primers used for PCR amplification.

Gene	Primer	Sequence	Application	Reference
***COI***	AAnd2+300	5’-TCC ACA TCA TTA ATT CTT ATA GCC CTC C-3’	Long PCR primers for *COI* of *A*. *alayseae*	[34]
	AAatp6-450	5’-AGC AAG TGT TCC TGG ACG AAT AAT G-3’		
	AACO1+sF	5’-TTT CTA CAA ATC ATA AAG ACA TTG G-3’	Sequencing primers for the *COI* barcoding region of *A*. *alayseae*	
	AACO1-sR	5’-AGC ATG GCG TAG ATC ATA CCT AGA G-3’		
	LCO1490	5'-GGT CAA CAA ATC ATA AAG ATA TTG G-3'	Universal primers for the *COI* barcoding region of *A*. *hourdezi* and *G*. *puia*	[42]
	HCO2198	5'-TAA ACT TCA GGG TGA CCA AAA AAT CA-3'		
***16S rDNA***	16Sa	5’-CGC CTG TTT ATC AAA AAC AT-3’	Universal primers for mitochondrial *16S rDNA*	[43]
	16Sb	5’-CTC CGG TTT GAA CTC AGA TCA-3’		
***28S rDNA***	28Sa	5’-GAC CCG TCT TGA AAC ACG GA-3’	Universal primers for nuclear *28S rDNA*	[44]
	28Sb	5’-TCG GAA GGA ACC AGC TAC TA-3’		

### Phylogenetic analysis

The new sequences obtained in this study were aligned with those of other bythograeids and the Atlantic blue crab *Callinectes sapidus*, used as an outgroup (S2 Table), which were retrieved from GenBank using the Geneious alignment method implemented in Geneious Prime, and adjusted through visual inspection. Intra- and interspecific variations in individual gene alignment were calculated using MEGA X [45] based on the p-distance value.

To construct a phylogenetic tree of bythograeid crabs, individual *COI*, *16S rRNA*, and *28S rRNA* gene alignments were concatenated to form a single multiple-sequence alignment using Geneious Prime. The best-fitting model of nucleotide substitution was then determined using the Akaike information criterion (AIC) in JModelTest 2.1.7 [46], and the model GTR + I + G was selected as the best evolution model. Next, maximum likelihood (ML) and Bayesian inference (BI) tests were performed using RAxML version 8.2.11 [47] and MrBayes 3.2.6 [48], respectively, implemented in Geneious Prime with the gene partition option. Confidence in the resulting bythograeid relationships was assessed based on the bootstrap proportion (BP) with 100 replications for the ML model. For the BI analysis, four Markov chain Monte Carlo chains were run for 1,000,000 generations and sampled every 200 generations. Bayesian posterior probability (BPP) values were estimated after the initial 500 (10%) trees were discarded as burn-in.

### Nucleotide diversity and haplotype network

The number of polymorphic sites, nucleotide diversity, number of haplotypes, haplotype diversity, Tajima’s *D*, Fu’s *F*_*S*_, and pairwise differences (*F*_*ST*_) were estimated using DnaSP v5.0 [49] and Arlequin v. 3.5 [50]. Significance levels for Tajima’s *D*, Fu’s *F*_*S*_, and pairwise *F*_*ST*_ were corrected using the Bonferroni method [51]. Analysis of molecular variance (AMOVA) was performed in Arlequin v. 3.5 [50] to detect the population differentiation of *COI* haplotypes. To determine the genetic relationships among hydrothermal vent field populations and *COI* haplotypes, median-joining networks [52] were created using Arlequin v. 3.5 [50] and graphed with PopArt v. 1.7 [53]. Migration rates, population sizes, and relative numbers of migrants were estimated using MIGRATE v. 4.4.0 [54].

## Results

### Phylogenetic position of *A*. *alayseae* within the bythograeid lineage

**Fig 2:**
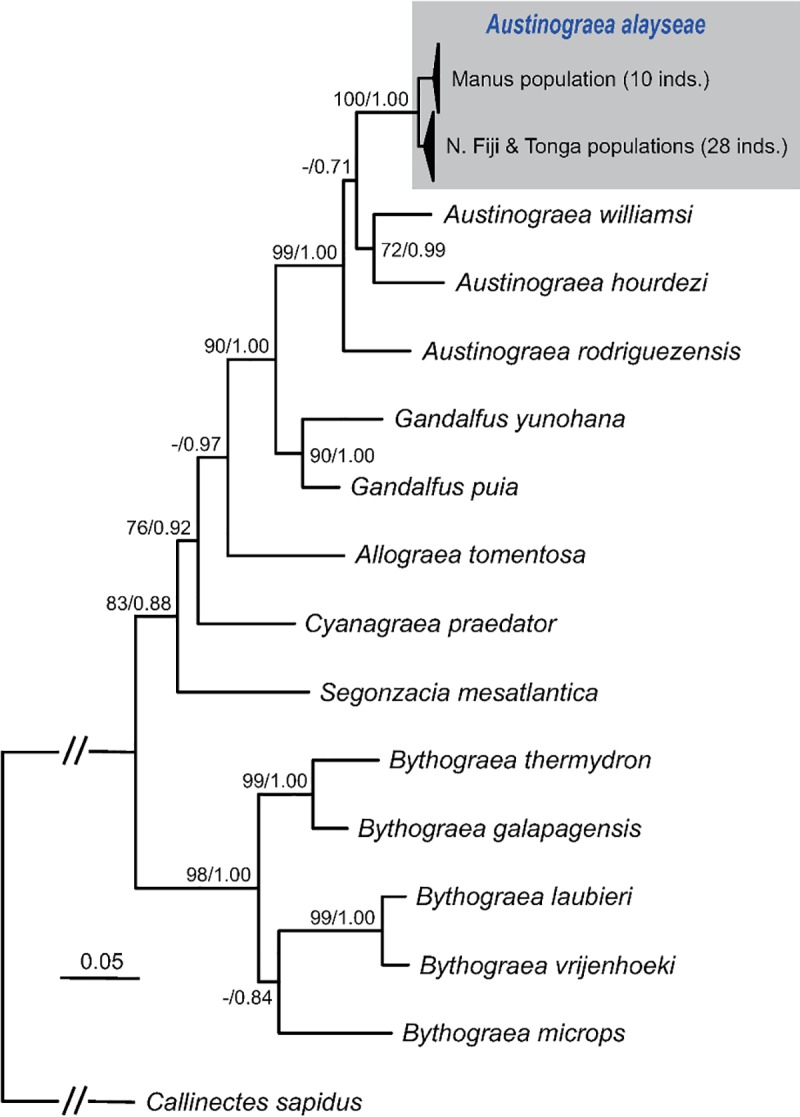
Phylogenetic tree of bythograeid crabs, constructed using a concatenated dataset of *COI*, *16S rRNA*, and *28S rRNA* genes. The gray-shaded box highlights three *Austinograea alayse*ae populations. Numbers at internodes are the maximum likelihood bootstrap proportions (left) and Bayesian posterior probabilities (right). Asterisks indicate bootstrap values <60%.

*Austinograea alayseae* is distributed widely across hydrothermal vent areas in the Southwest Pacific Ocean. In this study, we obtained the sequences of three genes, two mitochondrial genes (*COI* and *16S rDNA*) and one nuclear gene (*28S rDNA*), from 38 specimens of *A*. *alayseae*, which included 10 individuals from the Manus Basin (Manus population), 11 individuals from the North Fiji Basin (North Fiji population), and 17 individuals from the Tonga Arc (Tonga population). Then, phylogenetic trees of bythograeids were constructed using the concatenated sequences of the three genes (Fig 2; S1 Table). We could not include sequences of *A*. *jolliveti* and *B*. *intermedia* because they were not available from open-access sequence databases. The tree topologies were consistent with those obtained in previous studies [17, 34, 55]. Three genera, *Austinograea*, *Bythograea*, and *Gandalfus*, were each supported strongly as monophyletic taxa, with high support values of 99% BP and 1.00 BPP, 98% BP and 1.00 BPP, and 90% BP and 1.00 BPP, respectively. The newly added individuals of *A*. *alayseae* formed a monophyletic assemblage with 100% BP and 1.00 BPP, representing one of the most recently derived species in the bythograeid lineage. *Austinograea alayseae* individuals were separated into two clades, the Manus clade and the North Fiji–Tonga clade, which had very short branch lengths with respect to the collection region.

In terms of nucleotide sequence divergence, all *28S rDNA* sequences were identical among all three populations of *A*. *alayseae* (Table 4). The proportions of intraspecific variation in *16S rDNA* and *COI* were 0.00–1.69% and 0.00–4.08%, respectively. We observed the maximum variation between the Manus and North Fiji–Tonga clades (Table 5). In particular, the maximum value of *COI* variation was found in the middle of the barcoding gap, which is the gap between inter- and intraspecific variation, for bythograeids [34, 40].

**Table 4:** Intra- and interspecies variations in *COI*, *16S rDNA*, and *28S rDNA* sequences of Bythograeidae. Sequence variations were calculated from the nucleotide sequences using the p-distance method in MEGA X.

Group	Variation (%)					
	*COI*		*16S*		*28S*	
	Min.	Max.	Min.	Max.	Min.	Max.
Bythograeidae						
Intraspecies						
*Austinograea alayseae*	0	4.08	0	1.69	0	0
*A*. *hourdezi*	0	0	0	0.22	0	0
*A*. *rodriguezensis*	0	0.68	0	0.38	-	-
*Gandalfus puia*	0	0.51	0	0.38	0	0
*G*. *yunohana*	-	-	0	0	-	-
*Segonzacia mesatlantica*	-	-	0	0	-	-
*Cyanagraea praedator*	-	-	0.81	0.81	-	-
Interspecies						
*Austinograea* (4^†^)	10.93	12.08	4.73	7.54	0.42	1.69
*Bythograea* (5^†^)	6.62	16.13	0.62	7.48	0.31	3.94
*Gandalfus* (2^†^)	10.50	10.50	5.38	5.38	-	-
Among bythograeid genera (6^‡^)	12.22	17.17	8.71	14.85	0.66	7.45

†Number of species

‡Number of genera

**Table 5:** Variation among *COI* nucleotide sequences of the bythograeid crab *Austinograea alayseae* collected from three back-arc basins in the Southwest Pacific Ocean.

								Nucleotide diversity (%)			Pairwise *F_ST_*
Population		N	S	H	h	*D*	*F_S_*	Min	Max	Mean	
Intra-population	Manus	10	8	7	0.87 ± 0.107	-1.87^a^	-4.04^a^	0.00	0.68	0.27	-
	N. Fiji	11	9	8	0.93 ± 0.067	-0.62	-3.46^a^	0.00	1.19	0.44	-
	Tonga	17	9	9	0.87 ± 0.068	-1.18	-4.41^a^	0.00	0.68	0.30	-
	N. Fiji–Tonga	28	13	13	0.90 ± 0.036	-1.24	-6.91^a^	0.00	0.47	0.27	-
	Overall	38	34	20	0.94 ± 0.022	0.76	-2.18	0.80	2.54	1.67	-
Inter-population	Manus and N. Fiji	-	-	-	-	-	-	3.23	4.08	3.70	0.90*
	Manus and Tonga	-	-	-	-	-	-	3.23	4.08	3.65	0.92*
	N. Fiji and Tonga	-	-	-	-	-	-	0.00	1.02	0.37	0.00

N, sample size; S, polymorphic sites; H, total number of haplotypes; h, haplotype diversity; D, Tajima’s D; F, Fu’s *F_S_*; *F_ST_*, F-statistic.

^a^ indicates statistically significant values at α = 0.017 after Bonferroni correction.

### Population genetic divergence of *A*. *alayseae*

We examined whether the differences between these two *A*. *alayseae* clades represented genetic divergence at the population level. Alignment of the *COI* sequences of *A*. *alayseae* allowed detection of 32 variable nucleotide sites at the third position and two sites at the first position in the codons. The degree of intrapopulation variation ranged from 0.00% to 1.19%, and the maximum divergence was found in the North Fiji population (Table 5). The degree of interpopulation variation between the Manus population and the other two regions was 3.23–4.08%, and the maximum value for divergence between the North Fiji and Tonga populations was only 1.02%, within the range of intrapopulation variation among the three populations. In addition, based on pairwise comparison of *F_ST_*, the Manus population differed significantly from the North Fiji and Tonga populations, whereas no difference was found between the North Fiji and Tonga populations.

Based on the variable sites in the *COI* sequences of *A*. *alayseae*, we examined 20 haplotypes, which showed an overall haplotype diversity of 0.94 (± 0.022). The Manus, North Fiji, and Tonga populations consisted of seven, eight, and nine haplotypes, respectively. All three populations showed relatively high (>0.8) degrees of haplotype diversity. The highest diversity value (0.93 ± 0.067) was obtained for the North Fiji population. Four haplotypes were identified in both the North Fiji and Tonga populations, whereas none were shared between the Manus and North Fiji–Tonga populations. According to the AMOVA, haplotype variation between the Manus and North Fiji–Tonga populations was greater than that between North Fiji and Tonga populations and those among individuals within each population (90.88% vs. 0.00% and 9.12%, respectively), suggesting genetic isolation of *A*. *alayseae* in the Manus and North Fiji–Tonga hydrothermal vent field regions. On the other hand, based on migration rates and population sizes, the estimated number of migrants between North Fiji and Tonga indicated bidirectional flow of *A*. *alayseae* from North Fiji to Tonga (4289 migrants/generation) and from Tonga to North Fiji (4467).

**Fig 3:**
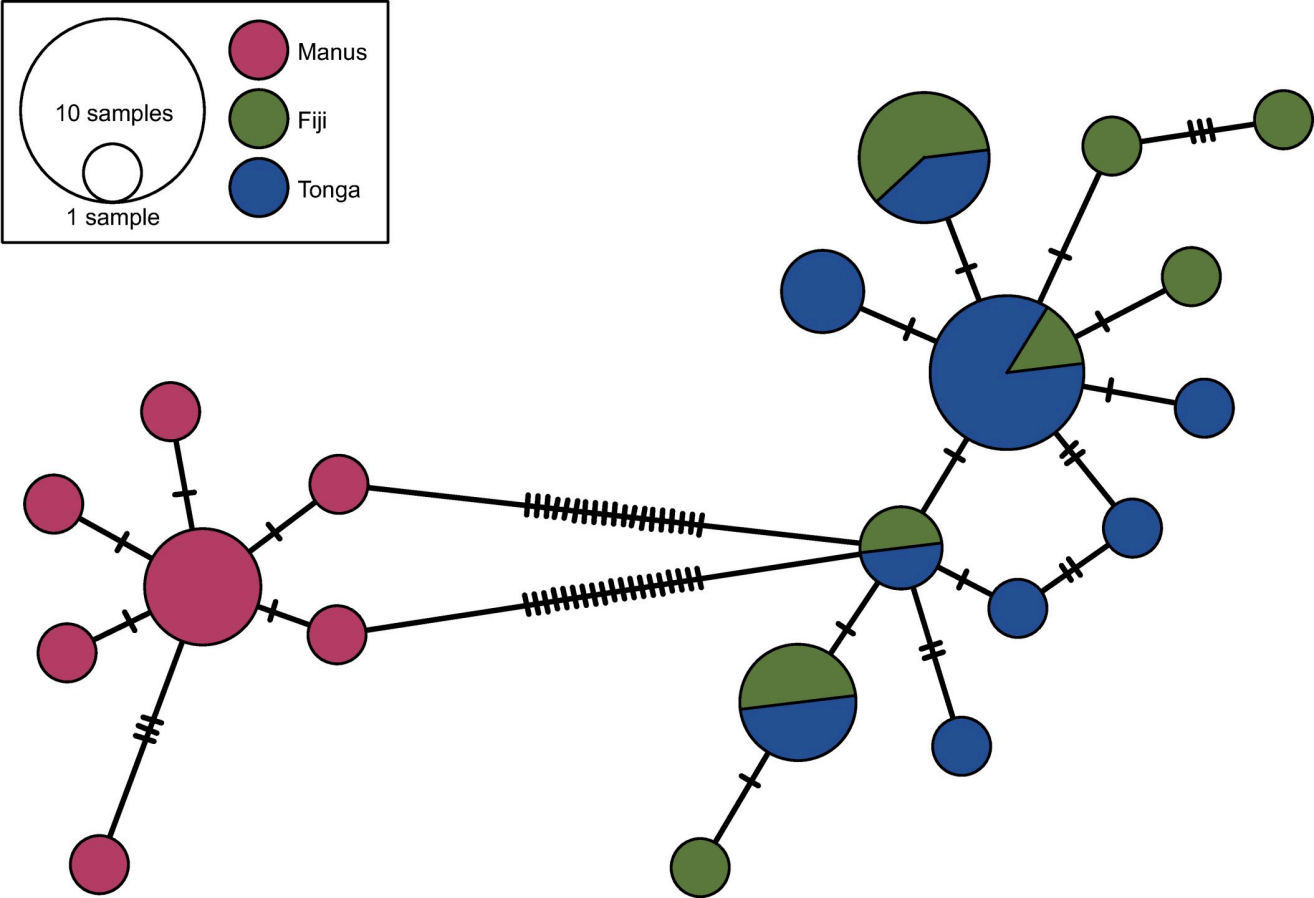
Median-joining network based on *COI* haplotypes from the bythograeid crab *Austinograea alayseae* collected from hydrothermal vent fields in the Southwest Pacific Ocean. Circle size and color reflect haplotype frequency and vent field, respectively. The number of short bars on each branch indicates the number of nucleotide substitutions between haplotypes.

In the haplotype network, the *COI* haplotypes of *A*. *alayseae* were divided distinctly into two clades, the Manus clade and the North Fiji–Tonga clade, with 14 nucleotide substitutions (Fig 3), which well reflects the phylogenetic relationship inferred using three genes (Fig 2). This result indicates that at some time in the past, *A*. *alayseae* living in the Southwest Pacific Ocean might have experienced a strong population bottleneck, which influenced separation of the two clades (Fig 2; Table 5). Since then, both clades have undergone independent population expansion (Tajima’s *D* and Fu’s *F_S_* < 0).

### Population genetic structure of invertebrates in the Southwest Pacific Ocean

Among vent organisms distributed evenly around hydrothermal vent fields in the Southwest Pacific Ocean, the vent crab *A*. *alayseae*, vent shrimp *Chorocaris* sp. 2, and black snail *Ifremeria nautilei* exhibit distinctive population genetic structures over a certain spatial scale or distance (Table 1) [7, 25]. The population genetic structure of *A*. *alayseae* recorded in this study demonstrated such distinct distribution patterns, with two segregated populations formed in the Manus and North Fiji–Tonga regions. This distribution is similar to those of *Chorocaris* sp. 2 and *I*. *nautilei*. However, whereas the populations of *Chorocaris* sp. 2 and *I*. *nautilei* share intermediate haplotypes, *A*. *alayseae* does not share any intermediate haplotype between clades (Fig 3). Based on this difference among the three species, we inferred that the *A*. *alayseae* clades were generated through a stepwise mutation process over a long period of time, with the possibility that intermediate haplotypes became extinct.

## Discussion

Previous studies have confirmed the monophyly of bythograeid crabs [17], which are distinguished from other Brachyura by reduction of the eyes at the adult stage and complete adaptation to hydrothermal vent environments [56]. Based on phylogenetic analysis, bythograeid crabs have been divided into two main groups, the *Bythograea* clade and a clade composed of *Austinograea*, *Gandalfus*, *Allograea*, *Cyanagraea*, and *Segonzacia* [17]. However, the established phylogenetic trees were constructed using specimens from a single vent area for each species, although some species (*A*. *alayseae*, *A*. *hourdezi*, *A*. *rodriguezensis*, *S*. *mesatlantica*, *B*. *laubieri*, and *B*. *thermydron*) are known to have wide distributions among hydrothermal vent fields. Genetic divergence analysis of bythograeids at the population level has been performed for only one species, *A*. *rodriguezensis*, and revealed no genetic differentiation between populations at two vent fields separated by 700 km (Central Indian Ridge) [55, 57].

In the *Bythograea* clade, *B*. *laubieri* and *B*. *vrijenhoeki*, which co-occur on the Southeast Pacific Rise, are the most recently diverged species [31]. These sympatric species have relatively little genetic difference (6.6%) in their *COI* sequences, which is the minimum interspecific variation observed in the family Bythograeidae (Table 4) [40]. In this study, populations of *A*. *alayseae* showed relatively large degrees of intraspecific variation (3.23–4.08%) between the Manus and North Fiji–Tonga clades. An analysis of bythograeids excluding *A*. *alayseae* revealed <1% intraspecific variation and 6.62–16.13% interspecific variation (Table 4). Although the proportion of variation between the *A*. *alayseae* clades is closer to the interspecific variation patterns of other bythograeids, we could not identify any morphological difference based on the original descriptions of *Austinograea* [29, 33]. In many cases, genetic differentiation among related species in recently diverged lineages is not reflected in morphological traits, which occasionally leads to taxonomic ambiguity [58–60]. After experiencing a population bottleneck, the original *A*. *alayseae* population in the Southwest Pacific Ocean could have experienced independent population expansion and genetic differentiation of the two *A*. *alayseae* clades. These findings for *A*. *alayseae* raise the possibility of incipient speciation, despite the current absence of morphological traits distinguishing the clades. In addition, Guinot and Segonzac (2018) [33] noted the presence of the invalid *Austinograea* species, *Austinograea* sp. aff. *A*. *alayseae*, in the Manus Basin. Thus, we cannot rule out the possibility that our specimens from the Manus Basin may be *Austinograea* sp. aff. *A*. *alayseae* which currently does not have confirmed taxonomic status (a new species or subspecies) due to insufficient morphological information and unavailable DNA sequences in public databases.

Larval dispersal is a main driver of gene flow and distribution of vent invertebrates, and it is related to local- and regional-scale geological and hydrological barriers, species-specific development processes, dispersal depth, and water temperature [9, 11, 12]. According to the modeling of potential larval dispersal distances with a planktonic larval duration of 83 days in the Southwest Pacific Ocean, a bidirectional connection was suggested only between North Fiji and New Hebrides (500 km apart), with an event of dispersal between these regions occurring once every 5,000–12,000 years [15]. Even with an increased planktonic larval duration of 170 days, the model did not assure a bidirectional connection between the Manus and North Fiji basins. Moreover, based on previous studies of the bythograeid crab *B*. *thermydron* and other species [61–63], the estimated duration of *A*. *alayseae* larval development does not exceed 3 months, which is too short for dispersal over a sufficient distance.

To elucidate the genetically differentiated clades of *A*. *alayseae*, geological and hydrographic barriers in the Southwest Pacific Ocean were also considered. Previous studies of geological features have led researchers to propose that the Vanuatu and New Guinea archipelagos, Solomon Islands, and New Hebrides, which are relatively young and not fully developed back-arc basins, acted as barriers or stepping stones, forming a genetic connection between the Manus and North Fiji basins [7, 15, 16]. Although we did not observe intermediate haplotypes between the Manus and North Fiji–Tonga clades, considering the recent differentiation of these clades, such intermediates may exist in newly formed vent regions. On the other hand, to explain the connection between the North Fiji and Tonga populations, we considered the theory that these volcanic areas originated from the disruption of a single arc <10 Mya [18]. Based on the estimated divergence times of decapod species [64] and our results, the divergence of the North Fiji–Tonga clade correlates with this geological event. Thus, the wide area of vent fields ranging from North Fiji to the Tonga/Lau region functioned as a geographical boundary, leading to reproductive isolation within the region and restricted gene flow from outside. In addition, the water masses in the North Fiji and Lau/Tonga regions are well mixed by the South Equatorial Current system [15, 65]. This mixing might have enabled bidirectional migration of *A*. *alayseae* between the North Fiji Basin and Tonga Arc. After larval dispersal, *A*. *alayseae* could have settled quickly and reliably in these regions, which have similar environmental features [12, 18].

Many questions remain unanswered concerning the origin and evolution of vent fauna in short-lived patchy habitats that last no more than a few decades. To improve our understanding of evolutionary processes, further research should be considered, including biogeographic analysis of additional taxa, investigation of mutation rates and average generation times of species over a geological time scale, and examination of the roles of other environmental features and life-history attributes, as well as genome-based population studies. Furthermore, to elucidate the gene flow of hydrothermal vent fauna between basins, we should investigate additional specimens of known species from unstudied regions throughout the Southwest Pacific Ocean.

## Supporting information

S1 Table Information about A. alayseae specimens used in this study. Classification, sampling locations, sample IDs, and DNA sample IDs of the species and GenBank accession numbers of genetic sequences used in this study.
(XLSX)

S2 Table Information about Bythograeidae specimens used for phylogenetic analysis. Classification and sampling locations of the species and GenBank accession numbers of genetic sequences used.
(XLSX)

S3 Table Information about Bythograeidae specimens used for distance calculation. Classification and sampling locations of the species and GenBank accession numbers of the *COI* gene sequences used.
(XLSX)

## References

[1] Van Dover CL, Reynolds GT, Chave AD, Tyson JA. Light at deep‐sea hydrothermal vents. Geophys Res Lett. 1996;23(16):2049–52.

[2] Corliss JB. Oases of life in the cold abyss. Natl Geogr Mag. 1977;152:441–53.

[3] Corliss JB, Dymond J, Gordon LI, Edmond JM. On the Galapagos Rift. Science. 1979;203:16. 17776033 10.1126/science.203.4385.1073

[4] Tunnicliffe V. The biology of hydrothermal vents: ecology and evolution. Ann Rev Mar Sci. 1991;29:319–407.

[5] German CR, Ramirez-Llodra E, Baker MC, Tyler PA, ChEss Scientific Steering Committee. Deep-water chemosynthetic ecosystem research during the census of marine life decade and beyond: a proposed deep-ocean road map. PLoS ONE. 2011;6(8):e23259 10.1371/journal.pone.0023259 21829722 PMC3150416

[6] Bickford D, Lohman DJ, Sodhi NS, Ng PK, Meier R, Winker K, et al Cryptic species as a window on diversity and conservation. Trends Ecol Evol. 2007;22(3):148–55. 10.1016/j.tree.2006.11.004 17129636

[7] Thaler AD, Plouviez S, Saleu W, Alei F, Jacobson A, Boyle EA, et al Comparative population structure of two deep-sea hydrothermal-vent-associated decapods (*Chorocaris* sp. 2 and *Munidopsis lauensis*) from southwestern Pacific back-arc basins. PLoS ONE. 2014;9(7):e101345 10.1371/journal.pone.0101345 24983244 PMC4077841

[8] Johnson SB, Warén A, Tunnicliffe V, Dover CV, Wheat CG, Schultz TF, et al Molecular taxonomy and naming of five cryptic species of *Alviniconcha* snails (Gastropoda: Abyssochrysoidea) from hydrothermal vents. Syst Biodivers. 2015;13(3):278–95.

[9] Van Dover CL. Biogeography of hydrothermal vent communities along seafloor spreading centers. Trends Ecol Evol. 1990;5(8):242–6. 10.1016/0169-5347(90)90063-J 21232364

[10] Tunnicliffe V, Fowler CMR. Influence of sea-floor spreading on the global hydrothermal vent fauna. Nature. 1996;379(6565):531–3.

[11] Desbruyères D, Hashimoto J, Fabri MC. Composition and biogeography of hydrothermal vent communities in Western Pacific back‐arc basins. Back-arc spreading systems: geological, biological, chemical, and physical interactions. Geophysical Monograph Series. 2006;166:215–34.

[12] Watling L, Guinotte J, Clark MR, Smith CR. A proposed biogeography of the deep ocean floor. Prog Oceanogr. 2013;111:91–112.

[13] Bachraty C, Legendre P, Desbruyères D. Biogeographic relationships among deep-sea hydrothermal vent faunas at global scale. Deep Sea Res Part 1 Oceanogr Res Pap. 2009;56(8):1371–8.

[14] Rogers AD, Tyler PA, Connelly DP, Copley JT, James R, Larter RD, et al The discovery of new deep-sea hydrothermal vent communities in the Southern Ocean and implications for biogeography. PLoS Biol. 2012;10(1):e1001234 10.1371/journal.pbio.1001234 22235194 PMC3250512

[15] Mitarai S, Watanabe H, Nakajima Y, Shchepetkin AF, McWilliams JC. Quantifying dispersal from hydrothermal vent fields in the western Pacific Ocean. Proc. Natl. Acad. Sci. 2016;113(11):2976–81. 10.1073/pnas.1518395113 26929376 PMC4801315

[16] Thaler AD, Zelnio K, Saleu W, Schultz TF, Carlsson J, Cunningham C, et al The spatial scale of genetic subdivision in populations of *Ifremeria nautilei*, a hydrothermal-vent gastropod from the southwest Pacific. BMC Evol Biol. 2011;11(1):372. 22192622 10.1186/1471-2148-11-372 PMC3265507

[17] Mateos M, Hurtado LA, Santamaria CA, Leignel V, Guinot D. Molecular systematics of the deep-sea hydrothermal vent endemic brachyuran family Bythograeidae: a comparison of three Bayesian species tree methods. PloS ONE. 2012;7(3):e32066 10.1371/journal.pone.0032066 22403623 PMC3293879

[18] Auzende J-M, Lafoy Y, Marsset B. Recent geodynamic evolution of the north Fiji basin (southwest Pacific). Geology. 1988b;16(10):925–9.

[19] Galkin S. Megafauna associated with hydrothermal vents in the Manus Back-Arc Basin (Bismarck Sea). Mar Geol. 1997;142(1–4):197–206.

[20] Vrijenhoek RC. Genetic diversity and connectivity of deep‐sea hydrothermal vent metapopulations. Mol Ecol. 2010;19(20):4391–411. 10.1111/j.1365-294X.2010.04789.x 20735735

[21] Marsh L, Copley JT, Huvenne VA, Linse K, Reid WD, Rogers AD, et al Microdistribution of faunal assemblages at deep-sea hydrothermal vents in the Southern Ocean. PLoS ONE. 2012;7(10):e48348 10.1371/journal.pone.0048348 23144754 PMC3483289

[22] Plouviez S, Schultz TF, McGinnis G, Minshall H, Rudder M, Van Dover CL. Genetic diversity of hydrothermal-vent barnacles in Manus Basin. Deep Sea Res Part 1 Oceanogr Res Pap. 2013;82:73–9.

[23] Thaler AD. Population Genetics of Species Associated with Deep-sea Hydrothermal Vents in the Western Pacific. Ph. D. Dissertation, Duke University; 2012.

[24] Thaler AD, Saleu W, Carlsson J, Schultz TF, Van Dover CL. Population structure of *Bathymodiolus manusensis*, a deep-sea hydrothermal vent-dependent mussel from Manus Basin, Papua New Guinea. PeerJ. 2017;5:e3655 10.7717/peerj.3655 28852590 PMC5572536

[25] Kojima S, Segawa R, Fujiwara Y, Hashimoto J, Ohta S. Genetic differentiation of populations of a hydrothermal vent-endemic gastropod, *Ifremeria nautilei*, between the North Fiji Basin and the Manus Basin revealed by nucleotide sequences of mitochondrial DNA. Zoolog Sci. 2000;17(8):1167–74. 10.2108/zsj.17.1167 18522473

[26] Williams AB. A new crab family from the vicinity of submarine thermal vents on the Galapagos Rift (Crustacea: Decapoda: Brachyura. Proc Biol Soc Wash. 1980;93(2):443–72.

[27] Davie PJF, Guinot D, Ng PKL. Phylogeny of Brachyura. Treatise on Zoology—Anatomy, Taxonomy, Biology—The Crustacea, complementary to the volumes translated from the French of the Traité de Zoologie. 2015;9.

[28] Yang JS, Nagasawa H, Fujiwara Y, Tsuchida S, Yang WJ. The complete mitogenome of the hydrothermal vent crab *Gandalfus yunohana* (Crustacea: Decapoda: Brachyura): a link between the Bythograeoidea and Xanthoidea. Zool Scr. 2010;39(6):621–30.

[29] Hessler RR, Martin JW. *Austinograea williamsi*, new genus, new species, a hydrothermal vent crab (Decapoda: Bythograeidae) from the Mariana Back-Arc Basin, western Pacific. J Crustacean Biol. 1989;9(4):645–61.

[30] Tsuchida S, Hashimoto J. A new species of bythograeid crab, *Austinograea rodriguezensis* (Decapoda, Brachyura), associated with active hydrothermal vents from the Indian Ocean. J Crustacean Biol. 2002;22(3):642–50.

[31] Guinot D, Hurtado LA. Two new species of hydrothermal vent crabs of the genus Bythograea from the southern East Pacific Rise and from the Galapagos Rift (Crustacea Decapoda Brachyura Bythograeidae). C R Biol. 2003;326(4):423–39. 12876893 10.1016/s1631-0691(03)00126-4

[32] Martin JW, Haney TA. Decapod crustaceans from hydrothermal vents and cold seeps: a review through 2005. Zool J Linn Soc. 2005;145(4):445–522.

[33] Guinot D, Segonzac M. A review of the brachyuran deep-sea vent community of the western Pacific, with two new species of *Austinograea* Hessler & Martin, 1989 (Crustacea, Decapoda, Brachyura, Bythograeidae) from the Lau and North Fiji Back-Arc Basins. Zoosystema. 2018;40(1):75–107.

[34] Kim SJ, Lee KY, Ju SJ. Nuclear mitochondrial pseudogenes in *Austinograea alayseae* hydrothermal vent crabs (Crustacea: Bythograeidae): effects on DNA barcoding. Mol Ecol Resour. 2013;13(5):781–7. 10.1111/1755-0998.12119 23663201

[35] Won Y, Young C, Lutz R, Vrijenhoek R. Dispersal barriers and isolation among deep‐sea mussel populations (Mytilidae: *Bathymodiolus*) from eastern Pacific hydrothermal vents. Mol Ecol. 2003;12(1):169–84. 12492886 10.1046/j.1365-294x.2003.01726.x

[36] Beinart RA, Sanders JG, Faure B, Sylva SP, Lee RW, Becker EL, et al Evidence for the role of endosymbionts in regional-scale habitat partitioning by hydrothermal vent symbioses Proc Natl Acad Sci U. S. A. 2012:201202690. 10.1073/pnas.1202690109 PMC3511114 23091033

[37] Jang S-J, Park E, Lee W-K, Johnson SB, Vrijenhoek RC, Won Y-J. Population subdivision of hydrothermal vent polychaete *Alvinella pompejana* across equatorial and Easter Microplate boundaries. BMC Evol Biol. 2016;16(1):235 10.1186/s12862-016-0807-9 27793079 PMC5084463

[38] Guinot D. *Austinograea alayseae* sp. nov., Crabe hydrothermal découvert dans le bassin de Lau, Pacifique sud-occidental (Crustacea Decapoda Brachyura). Bull Mus Hist Nat Paris. 1990;4(11):879–903.

[39] Kim S-J, Kim HS, Ju S-J. Mitochondrial genome of the hydrothermal vent crab *Austinograea alayseae* (Crustacea: Bythograeidae): genetic differences between individuals from Tofua Arc and Manus Basin. Mitochondrial DNA. 2014. 10.3109/19401736.2013.800489 23795854

[40] Lee W-K, Ju S-J, Hou BK, Kim S-J. DNA Barcoding for the Hydrothermal Vent Crab *Austinograea* Species (Crustacea: Bythograeidae) from the North Fiji Basin, Southwestern Pacific Ocean. Anim Syst Evol Diversity. 2019;35(1):30–2.

[41] Kim SJ, Min GS. Optimization of DNA extraction from a single living ciliate for stable and repetitive PCR amplification. Anim Cells Syst. 2009;13(3):351–6.

[42] Folmer O, Black M, Lutz R, Vrijenhoek R. DNA primers for amplification of mitochondrial cytochrome c oxidase subunit I from diverse metazoan invertebrates. Mol Mar Biol Biotechnol. 1994;3(5):294–9. 7881515

[43] Palumbi S, Martin A, Romano S, McMillan WO, Stice L, Grabowski G. Simple fool's guide to PCR. University of Hawaii, Honolulu: Privately published, compiled by S Palumbi 1991.

[44] Whiting MF, Carpenter JC, Wheeler QD, Wheeler WC. The Strepsiptera problem: phylogeny of the holometabolous insect orders inferred from 18S and 28S ribosomal DNA sequences and morphology. Syst Biol. 1997;46(1):1–68. 11975347 10.1093/sysbio/46.1.1

[45] Kumar S, Stecher G, Li M, Knyaz C, Tamura K. MEGA X: Molecular Evolutionary Genetics Analysis across Computing Platforms. Mol Biol Evol. 2018;35(6):1547–9. 10.1093/molbev/msy096 29722887 PMC5967553

[46] Darriba D, Taboada GL, Doallo R, Posada D. jModelTest 2: more models, new heuristics and parallel computing. Nat Methods. 2012;9(8):772. 10.1038/nmeth.2109 PMC4594756 22847109

[47] Stamatakis A. RAxML version 8: a tool for phylogenetic analysis and post-analysis of large phylogenies. Bioinformatics. 2014;30(9):1312–3. 10.1093/bioinformatics/btu033 24451623 PMC3998144

[48] Ronquist F, Teslenko M, Van Der Mark P, Ayres DL, Darling A, Höhna S, et al MrBayes 3.2: efficient Bayesian phylogenetic inference and model choice across a large model space. Syst Biol. 2012;61(3):539–42. 10.1093/sysbio/sys029 22357727 PMC3329765

[49] Librado P, Rozas J. DnaSP v5: a software for comprehensive analysis of DNA polymorphism data. Bioinformatics. 2009;25(11):1451–2. 10.1093/bioinformatics/btp187 19346325

[50] Excoffier L, Lischer HE. Arlequin suite ver 3.5: a new series of programs to perform population genetics analyses under Linux and Windows. Mol Ecol Resour. 2010;10(3):564–7. 10.1111/j.1755-0998.2010.02847.x 21565059

[51] Rice WR. Analyzing tables of statistical tests. Evolution. 1989;43(1):223–5. 10.1111/j.1558-5646.1989.tb04220.x 28568501

[52] Bandelt H-J, Forster P, Röhl A. Median-joining networks for inferring intraspecific phylogenies. Mol Biol Evol. 1999;16(1):37–48. 10.1093/oxfordjournals.molbev.a026036 10331250

[53] Leigh JW, Bryant D. POPART: full-feature software for haplotype network construction. Methods Ecol Evol. 2015;6(9):1110–6.

[54] Beerli P. How to use MIGRATE or why are Markov chain Monte Carlo programs difficult to use. Population genetics for animal conservation. Cambridge University Press 2009;17:42–79.

[55] Beedessee G, Watanabe H, Ogura T, Nemoto S, Yahagi T, Nakagawa S, et al High connectivity of animal populations in deep-sea hydrothermal vent fields in the Central Indian Ridge relevant to its geological setting. PLoS ONE. 2013;8(12):e81570 10.1371/journal.pone.0081570 24358117 PMC3864839

[56] Leignel V, Hurtado L, Segonzac M. Ecology, adaptation and acclimatisation mechanisms of Bythograeidae Williams, 1980, a unique endemic hydrothermal vent crabs family: current state of knowledge. Mar Freshw Res. 2018;69(1):1–15.

[57] Chen C, Linse K, Copley JT, Rogers AD. The ‘scaly-foot gastropod’: a new genus and species of hydrothermal vent-endemic gastropod (Neomphalina: Peltospiridae) from the Indian Ocean. J Molluscan Stud. 2015;81(3):322–34.

[58] Barluenga M, Stölting KN, Salzburger W, Muschick M, Meyer A. Sympatric speciation in Nicaraguan crater lake cichlid fish. Nature. 2006;439(7077):719 10.1038/nature04325 16467837

[59] Lanzaro GC, Lee Y. Speciation in *Anopheles gambiae*—The distribution of genetic polymorphism and patterns of reproductive isolation among natural populations. In: S Manguin editor. Anopheles mosquitoes-New insights into malaria vectors. Rijeka: InTech; 2013 p. 173–96.

[60] Lamichhaney S, Berglund J, Almén MS, Maqbool K, Grabherr M, Martinez-Barrio A, et al Evolution of Darwin’s finches and their beaks revealed by genome sequencing. Nature. 2015;518(7539):371 10.1038/nature14181 25686609

[61] Van Dover CL, Factor JR, Williams AB, Berg CJ Jr. Reproductive patterns of decapod crustaceans from hydrothermal vents. Bull Biol Soc Wash. 1985;6:223–7.

[62] Epifanio C, Perovich G, Dittel A, Cary S. Development and behavior of megalopa larvae and juveniles of the hydrothermal vent crab *Bythograea thermydron*. Mar Ecol Prog Ser. 1999;185:147–54.

[63] Wu X, Zeng C, Southgate PC. Ontogenetic patterns of growth and lipid composition changes of blue swimmer crab larvae: insights into larval biology and lipid nutrition. Mar Freshw Res. 2014;65(3):228–43.

[64] Yang J-S, Lu B, Chen D-F, Yu Y-Q, Yang F, Nagasawa H, et al When did decapods invade hydrothermal vents? Clues from the Western Pacific and Indian Oceans. Mol Biol Evol. 2012;30(2):305–9. 10.1093/molbev/mss224 23002089

[65] Speer K, Thurnherr AM. The Lau Basin Float Experiment (LAUB-FLEX). Oceanography. 2012;25(1):284.

